# Mining Google Trends Data for Health Information: The Case of the Irish “CervicalCheck” Screening Programme Revelations

**DOI:** 10.7759/cureus.5513

**Published:** 2019-08-29

**Authors:** Paul M Ryan, C Anthony Ryan

**Affiliations:** 1 Internal Medicine: Diabetes and Endocrinology, University College Cork, Cork, IRL; 2 Pediatrics and Child Health, University College Cork, Cork, IRL

**Keywords:** cervicalcheck, smear test, hpv, google trends

## Abstract

Background

In April 2018, the Irish cervical smear screening programme, “CervicalCheck”, came under intense scrutiny as the accuracy of hundreds of “negative” results were brought in question.

Aim

The goal of this brief report was to assess the impact of this real-life event on public information-seeking behaviour, using Google search anomalies as a proxy. Irish relative search volume data for several terms relating to cervical testing/cancer and human papillomavirus were extracted for a five-year period from February 2014 to January 2019 and analysed for the presence of anomalous spikes and shifts in the mean baseline.

Results

An unprecedented positive spike in searches relating to cervical testing/cancer was observed immediately after the CervicalCheck revelations, which remained anomalous for the month to follow (*p* < 0.05). This public interest preceded a mirroring increase in uptake of complimentary consultations offered by the Department of Health to the women concerned. Despite this service engagement and interest in cervical health, the relative search volumes for terms “human papillomavirus infection” and ”HPV vaccine” were just 78 and 51% of their maximum search volume for the five-year period.

Conclusions

Anomaly analysis revealed an unprecedented spike in information-seeking behaviour following the CervicalCheck revelations. However, this was not associated with a comparable elevation in HPV interest. This suggests that more public education and promotion of the HPV vaccine is warranted, in the context of vastly reduced uptake in recent years. Finally, Google Trends data represents a free an open source means by which to assess information-seeking behaviour of the public in relation to health and disease.

## Introduction

“CervicalCheck” is the Irish national screening programme which allows women aged 25-60 years old to avail of free cervical smear test. The cytological analysis has been carried out by two Irish and two US laboratories since 2008. Until recently, the programme appeared to be functioning well and, as such, garnered little attention. In April 2018, however, it was revealed that roughly 220 women with cervical cancer were never informed that their negative smear test results were inaccurate. Subsequent inquiry into the revelations revealed that, although the laboratories contracted to perform the sample processing and testing were safe, there existed serious failures in governance and structure of the screening programme, including a grave lack of transparency with in ongoing performance audits which led to harmful delays in many diagnoses. This revelation and the lack of publicly available information combined to create a media frenzy, which further fuelled the considerable public anxiety around the issue.

Human papillomavirus (HPV) is found to be associated with virtually all cervical cancers and a significant proportion of sexually active individuals will come into contact with the virus at some stage [1]. In 2010, Ireland launched their school-based HPV vaccination programme for girls aged 12-13 years and initially enjoyed impressive compliance of 80%, with a peak of 86.9% in 2014-2015 [2]. This success was in a climate in which mothers were found to have minimal knowledge of HPV or the vaccine, which left space for anti-vaccination lobby groups to spread misinformation [3]. Regrettably, the influence of anti-vaccination lobby groups on parental choice became a considerable issue in 2016, resulting in a drop to ~50% compliance rate.

Google Trends search data has proven to be a valuable complementary tool in epidemiological and public health research in recent years. Most recently, Google search data was used to demonstrate the increase in sexual assault and sexual harassment-related information-seeking behaviour in the wake of the #MeToo movement [4].

The present brief report aimed to utilise Irish Google search trends in the wake of the CervicalCheck revelations as a proxy for public interest. The authors predicted that Google searches relating to cervical testing and cancer would increase anomalously. In concert, we expected that the increased public fear would, in turn, lead to increased information-seeking behaviour relating to primary prevention of cervical cancer, that is, the HPV vaccine.

## Materials and methods

Data acquisition

This study monitored the volume of Google searches relating to the cervical smear test and HPV in Ireland over a five-year period (02/02/2014-20/01/2019). Data were extracted from the Google Trends website in January 2019 (https://trends.google.com/trends/). Initially the term “cervical check” was investigated and several of the top related queries were then included in the analysis. These additional terms included “cervical smear test” and “cervical cancer”. In addition, trend data for “HPV vaccine” and “human papillomavirus infection” searches were assessed over the same time period. Google provides relative search volumes (RSV), which outline the percentage proportion of the highest search volume in the predetermined region and period. Although this limits the comparability of the data to other studies, it is entirely adequate for the purposes of the present study as it is focussed solely in the presence of positive peaks, or anomalies. Finally, General Practitioner (GP) complimentary consultation data in the aftermath of the CervicalCheck revelations was garnered from the CervicalCheck Steering Committee weekly reports to the Minister for Health [5].

Statistical analysis

Data were imported to R through RStudio (v1.1.463) and prepared for analysis. R packages “Anomalize” (v0.1.1) and “Tidyverse” (v1.2.1) were used for anomaly analysis and plotting, respectively [6-7]. “Anomalize” allows the user to decompose time series, detect anomalies in the dataset and create bands separating the non-anomalous data from the anomalous spikes. In this analysis, an alpha of 0.05 was considered significant. In addition, GraphPad Prism (v6 for Mac, GraphPad Software, San Diego, California) was utilised for plotting of data.

## Results

**Figure 1:**
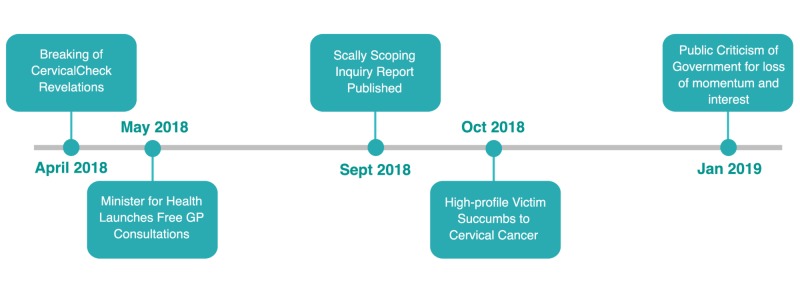
Major relevant events in the months following the disclosure of the CervicalCheck shortcomings GP, general practitioner

The simplified timeline of the CervicalCheck revelations is presented in Figure 1, demonstrating the initial reporting in April 2018 and subsequent commencement of general practitioner consultations the following month. Several additional points of significance are represented in the timeline, including publication of the Scally Scoping Inquiry Progress Report in September 2018. This was a thorough external investigation examining the events surrounding the CervicalCheck failures, including the standards of laboratory testing and internal governance of the programme itself, led by Dr. Gabriel Scally a Professor of Public Health at University of the West of England and the University of Bristol. Finally, the timeline includes the death of a high-profile victim in October 2018, as well as re-ignition of public discontent in January 2019 due to lack of progress.

**Figure 2:**
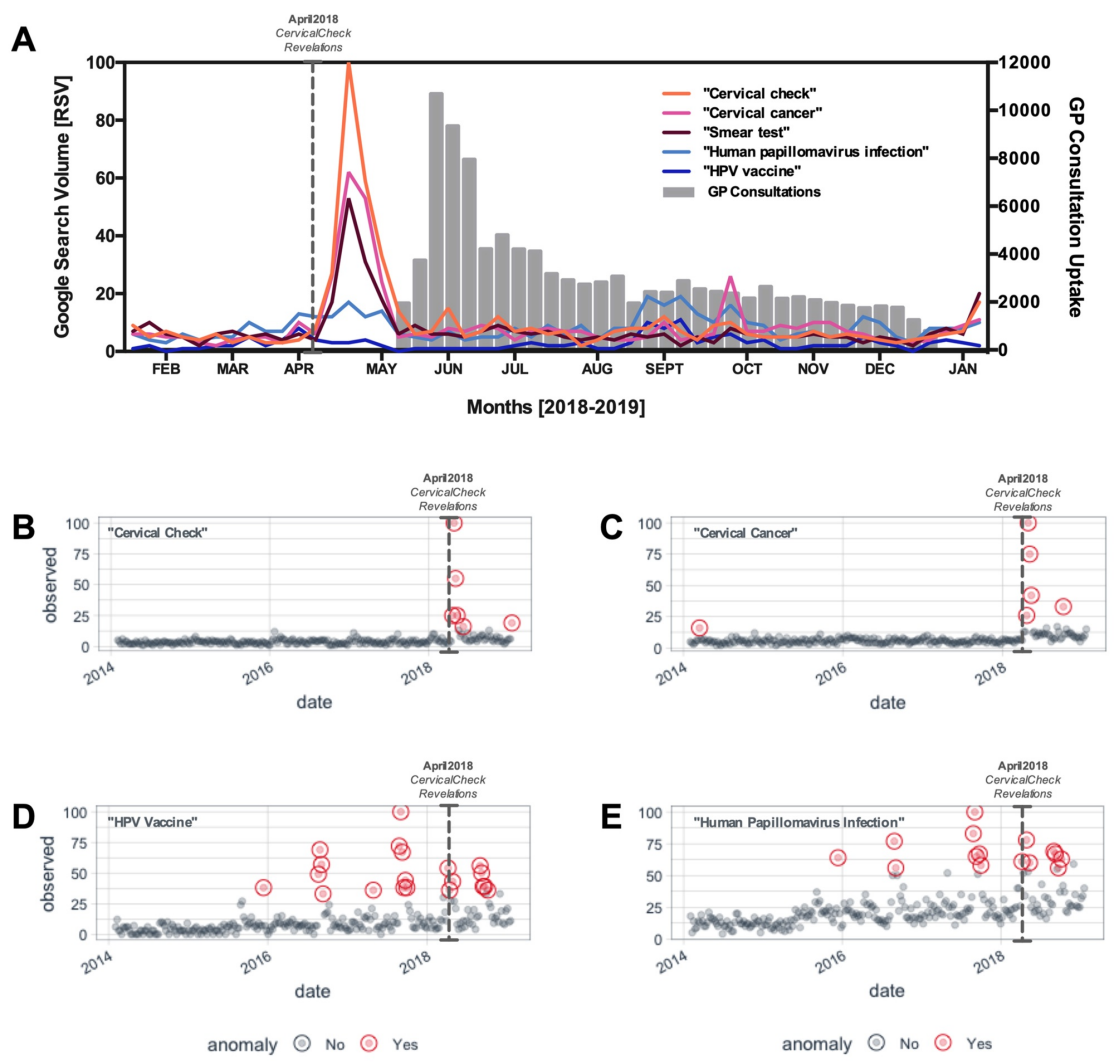
Anomaly analysis of cervical smear test-related Google searches and complimentary general practitioner visit uptake response [A] Detailed relative search volume of “cervical check” and commonly associated terms (lines), with reactionary uptake rates of free general practitioner consultations (bars). [B-E] Anomaly analysis of relative search volume for terms [B] “Cervical Check”, [C] “Cervical Cancer”, [D] "HPV Vaccine", [E] “Human Papillomavirus Infection", over the five year period (2014-2019 inclusive) with anomalies identified by red data points. Relative search volume is standardised to the highest search volume within the timeframe for each individual plot. RSV, relative search volume; GP, general practitioner.

Until recently, trends in Google searches relating to the CervicalCheck screening programme remained minimal, with modest annual non-anomalous peaks in January of each year. In response to the CervicalCheck failure disclosure, the Irish public reacted by vastly increasing the relative rate of Google searches with the terms “cervical check” and “cervical cancer” (p < 0.05; Figure 2A). This anomalous peak in interest was short-lived (4-5 weeks; Figures 2B-C), and interest in the terms did not reach anomalous heights again in the subsequent months. In turn, the Minister for Health announced complementary GP visits and cervical smear tests for any women concerned about their cervical screening. In line with this, almost 350,000 smear tests were ordered in Ireland in 2018, representing a ~40% increase in the annual testing rate (Figure 2A; grey bars).

Due to the considerable media attention afforded to the anti-vaccination lobby groups in previous years, we observed a number of anomalous search weeks for “HPV vaccine” (Figure 2D) and “human papillomavirus infection” (Figure 2E) from August 2015 onwards. Searches for both terms peaked in September 2017 and failed to reach similar levels thereafter (Figure 2A). Despite the substantial peak in cervical testing/cancer-related searches, “HPV vaccine” and “human papillomavirus infection” searches reached just 56% (Figure 2D) and 78% of maximum search volume (Figure 2E), respectively, in the period following the event. Furthermore, HPV related search terms were never found within the top related queries for terms “cervical check”, “cervical cancer” and “smear test” for data on the five-year period. This suggests that the Irish public did not seek information on HPV and cervical testing/cancer concurrently, and indicates that many may not entirely connect the two issues.

## Discussion

The CervicalCheck controversy was a public event which was high on emotion and low on reliable information. This, in turn, led to a media storm which propagated anxiety amongst the population, as is indicated by the results of this study. In January 2019, a high-profile victim publicly criticised the government’s management of the issue, suggesting that there was a loss of momentum and interest in reform. Indeed, the results of this study indicate that public attention for the issue dwindled relatively rapidly to baseline mean and did not reach comparable heights in the subsequent months, despite the publishing of an expert scoping inquiry into the failings of the CervicalCheck screening programme in September 2018 (Figure 1). However, the tragic loss of one of the acclaimed victims in October 2018 appears to stir moderate public interest, resulting in a single anomalous peak in “cervical cancer” searches (Figure 2A; pink line).

**Figure 3:**
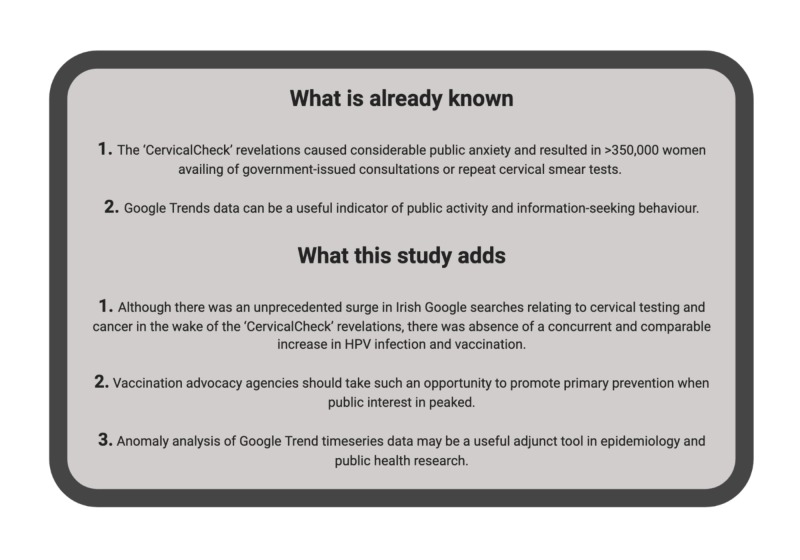
Current knowledge and report summary HPV, human papillomavirus

These data suggest that, although the CervicalCheck revelations triggered an increased information-seeking behaviour with regards to cervical testing and cancer, the Irish public did not demonstrate a heightened awareness of the primary prevention scheme for the main etiological contributor of the disease. The HPV vaccination programme has encountered challenging times in the past two years and, as a result, the HPV Vaccine Alliance was established in 2017 to present the facts about HPV vaccination in the factsheet and infographic-style outreach projects. Although efforts in educating the public appear to have stemmed the tide, the hangover from the anti-vaccination lobby groups is clear as uptake rates remain wholly inadequate. The present brief report suggests that vaccine advocacy groups should address the apparent understanding deficit and utilise high-profile publicity to reiterate the connection of HPV and cervical cancer to the public, in order to promote the national primary prevention scheme. This type of advocacy should be clear, factual and presented in a manner which does not detract from the suffering of those who were failed by the CervicalCheck programme. Importantly, Ireland has recently announced intentions to begin offering universal HPV vaccination for both males and females during the first year of secondary school education beginning in September 2019, representing a step forward with prospective protection which should not be understated. Previous knowledge and the conclusions of the present study are summarised in Figure 3.

Google Trends data is a publicly-available resource which appears to be a useful epidemiological and public health tool in assessing anomalous or seasonal information-seeking behaviour. The tool has recently been successfully applied to topics such as seasonality of several diseases, as well as public interest “cheap cigarettes” following the US states increases in cigarette taxation [8-11]. These cases demonstrate just a few manners in which such a dataset may be interrogated and demonstrate the versatility of this open-access resource. However, there are several important limitations to this brief report. Firstly, Google search data is an approximation of public interest and, while it does not represent the sole source of public information, it is a significant one. Secondly, due to the higher rates of internet users <65 years of age, this metric may be biased by youth over-sampling. Finally, the data released by Google Trends are not quantitative, but rather a representation of search volumes standardised relative to the highest search volume within the predetermined time and region. 

## Conclusions

In the wake of the CervicalCheck revelations, information-seeking behaviour regarding cervical testing and cancer was vastly increased in comparison to the previous five years. This anomalous spike in public interest immediately preceded a mirroring increase in uptake of complementary GP consultations offered by the Department for Health to concerned women. Despite this increase in cervical testing/cancer interest and healthcare engagement, we did not observe a comparable and concurrent increase in searches relating to HPV infection and vaccination. This indicates that the public currently may not conflate the two issues entirely and, therefore, further educational work is warranted to encourage primary prevention of HPV. Despite this, efforts are beginning to realise, with universal HPV vaccination being offered to secondary school attendees of both sexes in Ireland from September 2019 onwards. To our knowledge, this is the first study to use Google Trends data to examine public information-seeking behaviour in relation to cervical cancer testing and HPV, or the temporal association thereof.
